# Metagenomic Analysis of Surface Waters and Wastewater in the Colombian Andean Highlands: Implications for Health and Disease

**DOI:** 10.1007/s00284-024-04019-7

**Published:** 2025-02-28

**Authors:** Vanessa Urrea, Luisa Páez-Triana, Natalia Velásquez-Ortiz, Milena Camargo, Luz H. Patiño, Laura Vega, Nathalia Ballesteros, Arsenio Hidalgo-Troya, Luis-Alejandro Galeano, Juan David Ramírez, Marina Muñoz

**Affiliations:** 1 Centro de Investigaciones en Microbiología y Biotecnología -UR (CIMBIUR), Facultad de Ciencias Naturales, Universidad del Rosario, 110221 Bogotá, Colombia; 2 Centro de Tecnología en Salud (CETESA), Innovaseq SAS, 250027 Funza, Cundinamarca Colombia; 3 Grupo de Investigación Salud Pública, Departamento de Matemáticas y Estadística, Universidad de Nariño, 520002 Pasto, Colombia; 4 Grupo de Investigación en Materiales Funcionales y Catálisis (GIMFC), Departamento de Química, Universidad de Nariño, 520002 Pasto, Colombia; 5 Molecular Microbiology Laboratory, Department of Pathology, Molecular and Cell-Based Medicine, Icahn School of Medicine at Mount Sinai, New York, NY 10029 USA; 6 Instituto de Biotecnología-UN (IBUN), Universidad Nacional de Colombia, 111321 Bogotá, Colombia

## Abstract

Urban water bodies serve as critical reservoirs of microbial diversity, with major implications for public health and environmental quality. This study aimed to characterize the microbial diversity of surface waters and wastewater from the Pasto River in the Colombian Andean Highlands, offering insights that may support water quality monitoring efforts. Sampling was conducted at three river sites and one wastewater location. Standard physicochemical and microbiological analyses were performed, including real-time PCR to detect protozoan pathogens *Giardia* spp. and *Cryptosporidium* spp. Metagenomic sequencing provided an in-depth taxonomic and functional profile of microbial communities through two complementary approaches: (i) read-based analysis to identify abundant families and species, both pathogenic and beneficial, and (ii) detection of health-related molecular markers, including antimicrobial resistance markers and virulence factors. Physicochemical analyses showed distinct profiles between wastewater and surface water, with wastewater exhibiting elevated levels of suspended solids (113.6 mg/L), biochemical oxygen demand (BOD, 311.2 mg/L), and chemical oxygen demand (COD, 426.7 mg/L). Real-time PCR detected *Giardia* spp. DNA in 75% (76/102) of the samples and *Cryptosporidium* spp. DNA in 94% (96/102) of samples. The metagenomic read-based profiling identified *Aeromonas media* as a prevalent pathogen and *Polaromonas naphthalenivorans* as a potential biodegradative agent. The metagenomic assembly produced 270 high-quality genomes, revealing 16 bacterial species (e. g., *Acinetobacter johnsonii* and *Megamonas funiformis*) that provided insights into fecal contaminants and native aquatic microbes. Functional profiling further revealed a high prevalence of antimicrobial resistance markers, particularly for tetracyclines, aminoglycosides, and macrolides, with the highest abundance found in wastewater samples. Additionally, virulence factors were notably present in *Zoogloea ramigera.* The findings underscore the value of metagenomic profiling as a comprehensive tool for water quality monitoring, facilitating the detection of pathogens, beneficial species, and molecular markers indicative of potential health risks. This approach supports continuous monitoring efforts, offering actionable data for water management strategies to safeguard public health and maintain ecological integrity.

**Supplementary Information:**

The online version contains supplementary material available at 10.1007/s00284-024-04019-7.

## Introduction

Water is a vital resource that covers approximately 71% of the Earth's surface. However, only 2.5% of this water is freshwater [1], essential for humans, animals, and ecosystems [2]. Surface waters refer to freshwater bodies on Earth's surface [3], including rivers, lakes, and reservoirs. They are critical for sustaining human development and providing numerous social and economic benefits to urban growth. The increased water demand, especially in urbanized and developing regions, has led to significant pressures on these water bodies [4]. Pollution from urbanization, agriculture, industry, and domestic activities, among other anthropogenic activities, has contributed to the degradation of water quality, particularly pronounced in developing countries [5] where resources for implementing water source conservation and treatment schemes are limited. This contamination has contributed to ecological harm and the spread of waterborne diseases that affect human and animal health, ultimately limiting the sustainable use of freshwater resources [6].

Growing concerns about water resources underscore the urgent need for applying appropriate tests to evaluate the state of freshwater and effective wastewater treatment strategies that address both traditional and emerging contaminants. Pollutants in water bodies often originate from diverse sources, including domestic, hospital, and wastewater discharge, frequently containing personal care products, pharmaceuticals, and other potentially harmful substances [7]. For that reason, monitoring the physicochemical parameters of surface waters provides valuable insights into the impact of human activity on water quality.

Water bodies host a diverse range of microorganisms, including protozoa, bacteria, and viruses, some of which are associated with fecal contamination [9] and antibiotic resistance [10], thereby posing additional risks. For example, protozoan pathogens such as *Giardia* spp. and *Cryptosporidium* spp. serve as indicators of fecal contamination and are commonly associated with waterborne intestinal diseases that can negatively impact human health [11], especially among vulnerable populations. However, beyond these pathogens, water bodies harbor complex microbial communities that require detailed characterization to better understand ecosystem processes [17], functionality, adaptability, and the services they provide [18]. Characterizing these communities helps assess potential risks to human health, animal welfare, and ecological integrity [8]. Integrating physicochemical and microbiological parameters is crucial for a comprehensive view, as aquatic environments can promote the persistence and spread of contaminants to multiple areas [9–16], highlighting the need for continuous monitoring and assessment.

The advent of next-generation sequencing (NGS) technologies has significantly advanced microbiome analyses of environmental samples. Particularly, metagenomics has emerged as a powerful tool for characterizing microbial communities, allowing for taxonomic profiling of diverse microorganisms, including those that are difficult or impossible to culture. For example, Chopyk et al. conducted a study in the United States that analyzed microbial composition in freshwater and saline surface waters over time. Beyond taxonomic classification, metagenomics enables the identification of antibiotic resistance genes, enhancing the understanding of the risks associated with using these water bodies in activities like agriculture [19]. Similarly, a study in Southern China profiled pathogenic microorganisms and antibiotic resistance genes in wastewater and surface waters, finding similar microbial compositions in both environments suggesting that pathogens in surface waters might be linked to the direct discharge of untreated wastewater [20]. In Korea, metagenomic-assembled genomes (MAGs) from surface water provided detailed information into microbial community composition, resistome, and species without known cultivated representatives [21]. A Brazilian study underscored the importance of monitoring surface waters for pathogens, including protozoa like *Cryptosporidium* spp. and *Giardia* spp. to strengthen surveillance efforts [22]. In Mexico, NGS enabled the taxonomic profiling of surface waters, facilitating the identification of pathogenic microorganisms and their associations with water quality [23]. Additionally, research in wastewater revealed molecular markers relevant to public health, such as antibiotic resistance genes, highlighting the role of sewage and its treatment plants in identifying these genetic markers [24].

In Colombia, drinking water quality risk is assessed using the IRCA (Water Quality Risk Index), which evaluates physicochemical and microbiological characteristics. Based on the results, Colombia states (departments) are classified as high, medium, low, or no risk regarding water quality. The department of Nariño, located in Southwest Colombia, is classified as having a medium level of water quality risk according to the IRCA, and reports indicate related health issues in the region, including waterborne diseases [25]. Pasto River is one of the primary rivers in Nariño’s capital, supplies approximately 85% of the city's drinking water [26]. The river traverses three main basins with distinct uses: the upper basin supports agricultural activities, the middle basin flows through urban areas with significant domestic and industrial discharges, and the lower basin is predominantly used for irrigation [26–28]. Approximately, 90% of the city’s domestic wastewater is discharged into the river without treatment, with the Juan XXIII collector contributing substantial pollution loads in the northern region [27]. Despite these potential risks, only traditional microbiological methods have been used to assess the health hazards associated with the Pasto River.

The study aims to characterize the microbial communities in the surface and wastewater of the Pasto River, located in the Colombian Andean Highlands, to establish a baseline of microbial diversity and health-related molecular markers that can inform future monitoring and risk assessment strategies. Through traditional physicochemical analyses, real-time PCR detection of protozoan pathogens (*Giardia* spp. and *Cryptosporidium* spp.), and shotgun metagenomics, this research provides essential data for understanding the microbial composition and potential health risks associated with this water source. This study hypothesizes that surface waters and wastewater in the Pasto River contain diverse microbial communities with varying levels of pathogenicity and antimicrobial resistance, which could potentially impact public health, highlighting the need to employ these methods as a tool for monitoring water quality, supporting public health initiatives and environmental management strategies.

## Materials and Methods

### Sample Collection

This study was conducted on the Pasto River, the primary water resource for San Juan de Pasto, Colombia. Using EpiDat software (v.3.1) [29] for sample size calculation, we estimated the required samples based on an expected bacterial diversity of 79%, with a precision of 5% and a 95% confidence interval. This calculation was based on the research conducted by Köchling et al. [26], who collected samples from a river in north-eastern Brazil that was continuously exposed to industrial and domestic contaminants. These parameters required a minimum of five water samples for each sample type (surface water and wastewater). Over seven months from August 2022 to March 2023 (excluding January due to logistical issues), we collected 50 surface water samples from three locations—upper basin (S1), middle basin (S2), and lower basin (S3)—and 50 wastewater samples from the Juan XXIII wastewater collector (R4). This longitudinal sampling aimed to capture temporal shifts in microbiome composition across the four collection points.

Samples were collected using a plastic container attached to a 6-m rope, facilitating access to water bodies from bridges or the riverbank (Fig. S1-A). Before sampling, the container was rinsed with potable water and submerged multiple times in the surface water or wastewater to reduce contamination. For each point, two sampling methods were used: (i) single-sample collection, where 400 mL was collected at a specific time and location, and (ii) composite collection, which involved combining multiple sub-samples, obtaining 400 mL to represent basin-wide conditions. For surface water samples, the collection was obtained every 15 min, while for wastewater, 12 sub-samples were collected hourly. to ensure the representation of the basin (Fig. S1-B).

Finally, 102 samples were gathered, comprising 16 from point S1, 20 from point S2, 19 from point S3, and 47 from point R4, totaling 47 wastewater samples and 55 surface water samples. To preserve sample integrity and reduce microbial changes before processing, the samples were stored in sterile glass containers and placed in a portable refrigerator, maintaining a temperature of approximately 4 °C.

### Physicochemical Analysis of Surface and Wastewater Samples

Monthly aliquots of composite samples from S1 to R4 were analyzed for physicochemical parameters. *In-situ* measurements included pH, water and ambient temperatures, conductivity, and dissolved oxygen using a multiparameter instrument (HANNA instruments). Furthermore, turbidity, suspended solids, alkalinity, acidity, electrical conductivity, nitrites, chlorides, and phosphates were analyzed in a certified laboratory. Kruskal–Wallis test and Dunn’s test with Benjamini–Hochberg adjustment were conducted to highlight the particular months where these significant differences occurred. For this, we include significant differences * (0.01–0.05) and very significant differences **, *** (0.001–0.01, < 0.001).

### Sample Preprocessing and DNA Extraction

In the laboratory, biomass from the samples was concentrated through serial filtration using 3.0, 0.45, and 0.22 µm pore-sized membranes (Millipore). Each 100 mL portion of the sample was sequentially filtered, and membranes were subsequently placed in sterile 50 mL Falcon tubes. The obtained biomass was collected by gently scraping the membrane with molecular-grade water in two rounds of 15 min each. After centrifugation, DNA was extracted from the resulting pellet using the Qiagen DNeasy Power Soil kit, following the manufacturer's guidelines, with the elution buffer prewarmed to 65 °C for optimal yield. DNA quality and quantity were assessed using the Nanodrop equipment (Thermo Fisher Scientific), and integrity was confirmed via agarose gel electrophoresis.

### qPCR and Analysis for Protozoan Detection

Real-time PCR was employed to detect protozoan parasites *Giardia* spp. and *Cryptosporidium* spp. in water samples. The primer–probe sets used included GdF (5′-CATGCATGCCCGCTCA-3′), GdR (5′-AGCGGTGTCCGGCTAGC-3′), and GdP (6FAM/AGGACAACGGTTGCAC/MGB) for *Giardia* spp. with the following thermal profile: an initial 15-min phase at 50 °C, followed by 10 min at 95 °C. Subsequently, 40 cycles were carried out: 15 s at 95 °C, 1 min at 58 °C, and 1 min at 60 °C. CcF18S (5′-GTTTTCATTAATCAAGAACGAAAGTTAGG-3′), CcR18S (5′-GAGTAAGGAACAACCTCCAATCTCTAG-3′), and CsP (6HEX/TCAGATACCGTCGTAGTCTTAACCATAAACTATGCC/TAMRA) primers and probes were employed for the detection of *Cryptosporidium* spp. [26], with a thermal profile of 95 °C for 10 min, followed by 45 cycles (modified to enhance detection sensitivity): 10 s at 95 °C and 30 s at 60 °C.

### Metagenomic Sequencing and Data Analysis

Samples that met quality standards—average concentration > 40 ng/µL, 260/280 ratio between 1.8 and 2.0—were sent to Novogene for metagenomic sequencing. Targeting a minimum of 4 Gb raw reads per sample, sequencing was performed on an Illumina Novaseq platform using paired end reads of 150 bp.

After sequencing, reads were quality-checked using FastQC v.0.11.9 [30] and MultiQC v.1.6 [31]. Any adapter content, N content per base, or low-quality sequences were removed using Trimmomatic v.0.38 [32]. Taxonomic classification was performed using the Centrifuge software v.1.0.3-beta [33], with data visualization in Pavian [34] and RStudio. Additionally, analyses were conducted using eggNOG mapper v.2 [46], and metabolic pathways were elucidated using KofamKOALA [47] to explore functional aspects.

### Assembly of Genomes from Metagenomes

High-quality reads were assembled into Metagenomic-Assemble Genomes (MAGs) using Metaspades v3.15.3 [37]. Binning was then performed with MetaBAT, Maxbin, and Concoct software [38–40], followed by refinement using DASTool [41]. The quality of the MAGs was assessed with CheckM v1.1.3 [42], ensuring completeness > 90% and contamination < 5%. Taxonomic assignment for the MAGs was conducted using GTDB-Tk v.1.7.0 [43], while genome annotation was performed with Prokka [44] and visualized with Proksee (https://proksee.ca/).

A pangenome analysis was conducted using the Roary [45], generating a core genome alignment that served as the basis for a phylogenetic reconstruction that was visualized in the interactive Tree of Life—iToL (https://itol.embl.de/), to evaluate relationships among prominent microbiome members by comparison with publicly available reference genomes from BV-BRC, ensuring high completeness and quality in the comparisons.

### Identification of Health-Related Molecular Markers

Two analytical approaches were applied to detect molecular markers associated with antibiotic resistance (AMR-MM) and virulence factors (VF). The first approach used high-quality reads analyzed with the Resistance Gene Identifier (RGI) software, while the second approach was based on MAGs, using ABRICATE software. In both cases, the annotation of these markers was performed by comparison with the CARD v3.1.3 [35] and Virulence Factor Database (VFDB) [36] databases, respectively.

## Results

### Monitoring Physicochemical Parameters in Surface and Wastewater Samples

The traditional physicochemical parameters were subjected to a Shapiro–Wilks, revealing that only conductivity, dissolved oxygen percentage, pH, and water temperature exhibited a normal distribution. Other factors, such as fluorides, sulfates, and suspended solids, did not conform to this regular distribution pattern. We used the Kruskal–Wallis test for non-parametric data, which showed no significant differences across months. An ANOVA test indicated that significant differences were present only for the pH variable for parametric data. A subsequent Dunn’s test with Benjamini–Hochberg adjustment revealed notable variations in pH levels, specifically between March and November.

Despite the absence of statistically significant differences, a descriptive comparison was made between wastewater and surface water. Wastewater shows higher average water and ambient temperatures, with a difference of approximately 3.5 °C compared to surface water. Concerning phosphates, both wastewater (16.4 mg/L) and surface water (5.8 mg/L) samples exhibit elevated concentrations. In contrast, nitrites have a higher average value in surface water (0.2 mg/L) than in wastewater (0.1 mg/L). Suspended solids in wastewater (113.6 mg/L) exceed those in surface water (72 mg/L) by roughly 41.6 mg/L. Lastly, both BOD—Biochemical oxygen demand and COD—Chemical Oxygen Demand values are higher in wastewater (311.2 mg/L; 426.7 mg/L) in comparison to surface water (163.9; 269.4 mg/L) (Table S1).

### Detection of *Giardia* spp. and *Cryptosporidium* spp. in Surface and Wastewater Samples

Regarding the microbiological component, the genetic material of *Giardia* spp. and *Cryptosporidium* spp. was identified using real-time PCR. These protozoan parasites have been responsible for widespread gastrointestinal diseases, sometimes leading to fatalities, especially among children and individuals with weakened immune systems [48]. The DNA of *Giardia* spp. was detected in 76 out of 102 samples (75%), with 34 positive samples from wastewater and 42 from surface water. Samples without detectable *Giardia* spp. DNA was concentrated in three specific months. In November, six negative samples were obtained, two from the S3 surface water sampling point and four from the R4 wastewater point.

Another instance where *Giardia* spp. was not detected in specific samples collected in February. This month, 12 negative samples were distributed across all sampling points, with two at point S1, one at point S2, three at S3, and six at point R4. Finally, seven negative samples were identified in March, with two at S1, two at S3, and three at R4. Regarding detecting *Cryptosporidium* spp., it was found in 96 out of 102 samples (94%). Notably, the detection of *Cryptosporidium* spp. was higher throughout the study months, except for October (where one sample from the R4 wastewater point tested negative), November (with four negative samples at the R4 point), and March (with no detection at the S1 surface water sampling point).

### Description of the Aquatic Microbiome: A Taxonomic Profiling from Reads

Metagenomic analysis was conducted over 74/102 (72.5%) samples that successfully passed the sequencing quality control checks. Sequences were not trimmed, as they exhibited high-quality with scores exceeding 34 in phred score result, with an average of 33.4 million reads per sample. The analysis employed the entire dataset (Tables S2, S3, S4). A total of 52.8% of the reads were taxonomically classified using Centrifuge software. The sample composition was predominantly microbial, with bacteria accounting for over 98%. Most of the reads that did not classify as Bacteria corresponded to *Homo sapiens*, with a higher relative abundance in wastewater (0.60) compared to surface water (0.39). The most abundant archaea species in surface water samples were *Methanobrevibacter smithii* and *Methanococcus maripaludis*. *Methanosarcina barkeri* was the dominant species in wastewater. Lastly, the most abundant assigned viruses globally in the samples were *Aeromonas virus phiO18P* and *Acanthamoeba polyphaga mimivirus*. Notably, no reads were associated with protozoa or fungi from the reads. In pursuit of these, additional analyses were conducted, entailing the extraction of the 18S-rRNA region and subsequent comparison with the Kraken2 database [49]. However, these accounted for less than 0.08% of the total, prompting the decision not to pursue these analyses due to potential inaccuracies in taxonomic assignment.

### Temporal Analysis of Bacterial Family Dynamics in Wastewater and Surface Waters

**Fig. 1:**
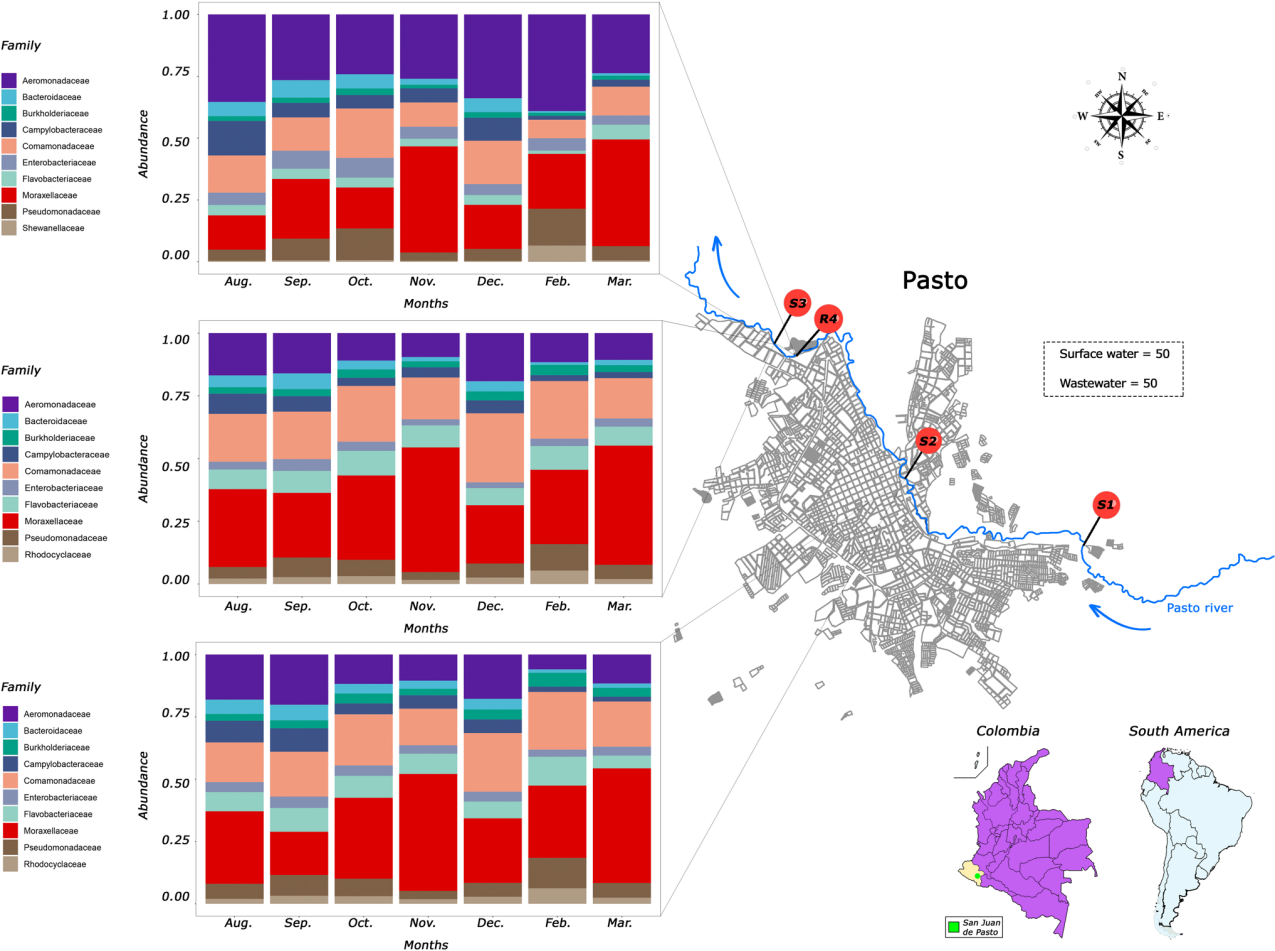
The top 10 most abundant bacterial families at the sampling points in the Pasto River. *S1* wastewater sampling point 1, *S2* surface water sampling point 2, *S3* surface water sampling point 3, *R4* wastewater collector point

We examined the prevalence of predominant bacterial families at each sample point along the San Juan de Pasto map. Figure 1 shows the locations: Point S1 is in the southern part of the city, Point S2 is in the central district, and Point S3 is in the northern region. The wastewater sampling point, R4, is also located in the north part of the city, upstream from Point S3, suggesting potential contamination from R4 point to S3 (Fig. 1). Comparisons were not performed for Site S1 due to the limited available samples (*N* = 1). To investigate potential variations among microbial families across different sampling months, we conducted a Kruskall–Wallis analysis.

No significant differences (*P*-value > 0.05) were observed for Site S2. However, differences did emerge between Sites S3 and R4. A Dunne test with Benjamini–Hochberg adjustment was conducted to highlight the specific months with significant differences. Site S3 showed significant differences for all families, as illustrated in Fig. S2. Variations within the Bacteroidaceae family were evident between February and September. Likewise, notable differences were identified in the Rhodocyclaceae and Pseudomonadaceae families between February and November (Fig. S2).

Moreover, variations in family composition were observed at wastewater point R4 across the months (Fig. S3). It is worth highlighting the significance of substantial differences, indicated by three asterisks (***) and characterized by P-value values below 0.001, as illustrated in Fig. S3. The Bacteroidaceae family exhibited significant differences, with a *P*-value of 0.002 when comparing September to February and 0.006 when comparing September to March. Additionally, variations were evident between October and February (*P*-value 0.009). The Burkholderiaceae family displayed variations in October compared to February (*P*-value 0.0006) and November (*P*-value 0.002). In contrast, the Campylobacteraceae family presented significant differences in February compared to August (*P*-value 0.00002) and December (*P*-value 0.00009). Similarly, the Comamonadaceae family showed variations between February and October (*P*-value 0.0002) and during August and February (*P*-value 0.001). The Enterobacteriaceae family revealed significant differences in March compared to October (*P*-value 0.0007) and between March and September (*P*-value 0.002). Concerning the Moraxellaceae family, changes in abundance were evident in October compared to December (*P*-value 0.008) and March (*P*-value 0.008). When examining changes in the Pseudomonadaceae family, significant differences were identified in November compared to February (*P*-value 0.0003) and October (*P*-value 0.00004). Lastly, the Shewanellaceae family, in addition to its distinctive profile relative to surface water points, exhibited variations in February and December (*P*-value 0.00008) and between February and November (*P*-value 0.00001).

### Abundant Bacterial Species with Pathogenic and Beneficial Traits

**Fig. 2:**
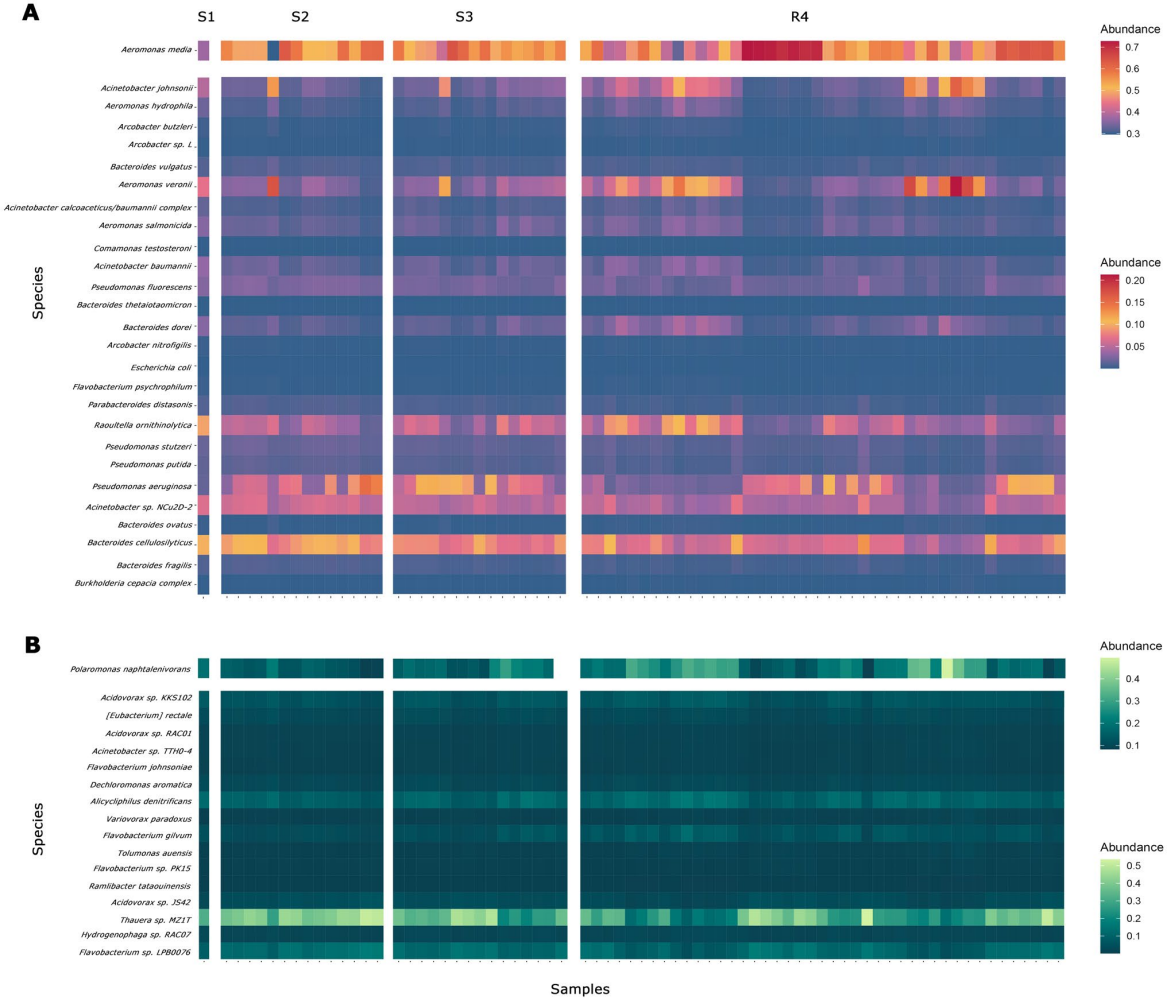
Global overview of the most abundant bacteria at all sampling points. **A** Bacteria associated with reported infections, **B** Bacteria with beneficial potential

The top 20 most abundant bacterial species were identified across sites, resulting in 44 highly abundant species shared among them. Figure 2A presents all the bacteria for which there are reports of being potentially pathogenic. Particularly abundant species include *Bacteroides cellulosilyticus, Raoultella ornithinolytica*, and *Pseudomonas aeruginosa*. *Aeromonas media* was the most abundant species in this classification. On the other hand, although the *Burkholderia cepacia* complex is not the most abundant, its presence is noteworthy among the top 44. Otherwise, beneficial bacteria included *Thauera* sp. MZ1T and *Alicycliphilus denitrificans* emerge as notable species. Lastly, *Polaromonas naphthalenivorans* was the most abundant bacterium in this classification (Fig. 2B).

### Assembly and Taxonomic Assignment from Metagenomes

Clean reads yielded 270 high-quality Metagenome-Assembled Genomes from (MAGs), meeting the Bowers et al. criteria of 90% completeness and < 5% contamination. Taxonomic assignment performed using GTDB-Tk allowed the acquisition of 175 MAGs classified at the species level, which were then compared to cross-references from PubMLST [50] to identify potential matches with known microorganism species. Reference genomes corresponding to each assigned species were visualized by comparison with publicly available genomes identified in BV-BRC: Bacterial and Viral Bioinformatics Resource Center [51] as a step to those genomes. Our selection criteria prioritized public genomes that were complete and high-quality. Species that lacked available reference genomes were excluded from further analyses.

Among assigned genomes, 50 originated from wastewater and 19 from surface water, representing 16 bacterial species. Prominent species included *Acinetobacter johnsonii, Megamonas funiformis* and *Phocaeicola vulgatus*.

### COG Functional Analysis and Pangenome Insights

**Fig. 3:**
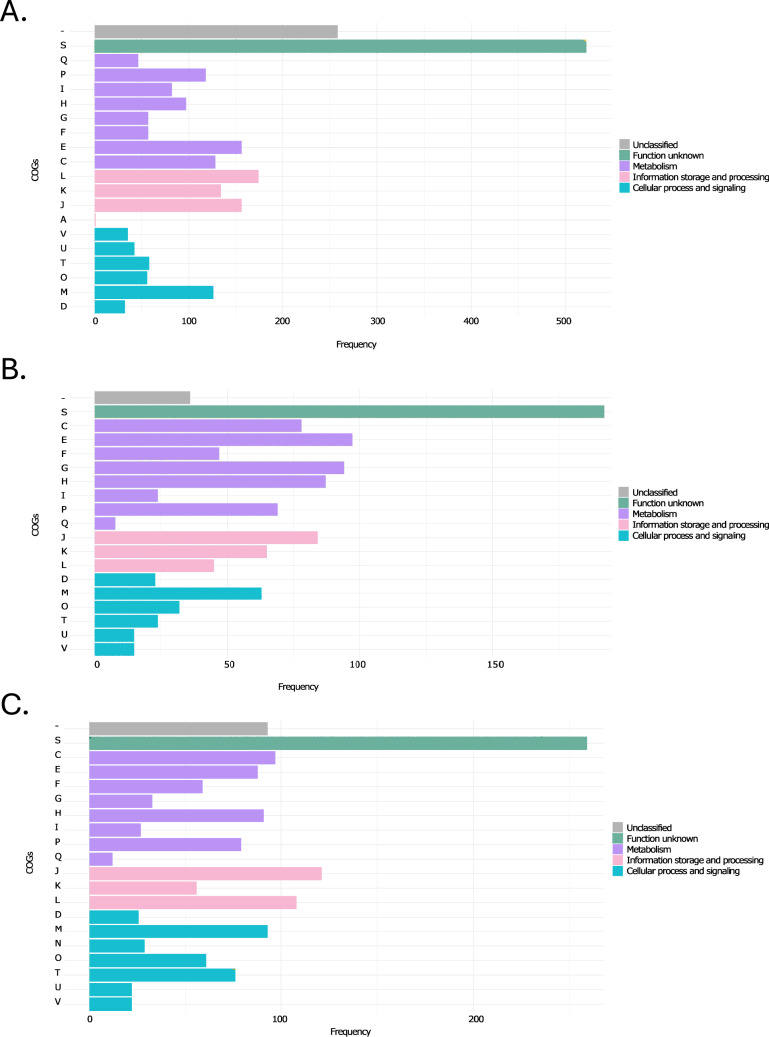
Clusters of Orthologous Groups (COGs) for the predominant species. **A** *Acinetobacter johnsonii*, **B** *Phocaeicola vulgatus*, and **C** *Megamonas funiformis*

Clusters of Orthologous Groups (COGs) analysis provided insights into the metabolic and functional diversity within predominant MAGs (Fig. 3). This overview highlights the notably high frequency within the "Function unknown" category. Moreover, across all samples, there was a more extensive range of subcategories within the overarching "Metabolism" category. For *A. johnsonii* displayed in the first panel of the figure, the COGs L and J from the information storage and processing category were the most prevalent. *P. vulgatus* also consistently presented COG M from the cellular process and signaling category. Moreover, *M. funiformis* shown in the third panel of the figure showed that within the COGs, the category "Metabolism" predominated over the others.

**Fig. 4:**
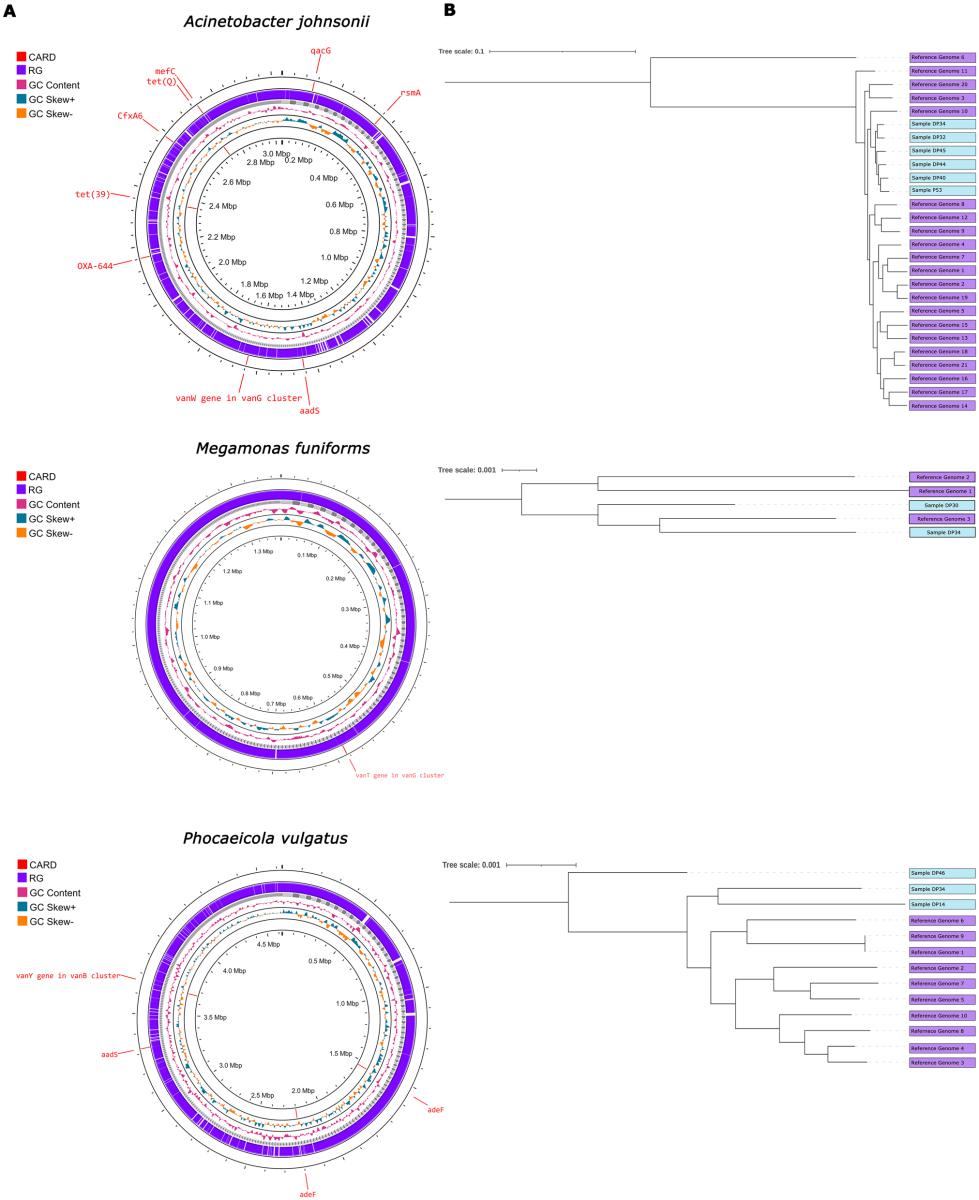
Phylogenetic reconstruction based on core genome alignment of the predominant Metagenome-assembled genomes. **A** *Acinetobacter johnsonii*, **B** *Phocaeicola vulgatus*, and **C** *Megamonas funiformis*

The genomes of the predominant MAGs species were analyzed alongside previously described reference genomes. For this analysis, we included MAGs with more than one bin and more than one assigned reference genome. The comparisons were visually represented using Proseek (Fig. 4A), and then midpoint-rooted trees for each species from core genome of each species were generated (Fig. 4B). Reference genomes are marked in purple (to access these, please refer to the following link in Table S5). In contrast, the studied genomes are marked in blue. Regarding *Acinetobacter johnsonii*, all study genomes clustered within the same clade. Interestingly, the reference genome did not cluster with the other reference genomes or ours. For *Megamonas funiformis*, most study genomes formed a distinct cluster. Lastly, in the case of *Phocaeicola vulgatus*, two of our genomes were closely grouped. In contrast, the remaining genome (Sample DP46) was the most distant and distinct compared to the other genomes, including the reference genomes.

### Relative Abundance of Molecular Markers of Health Interest from Reads

**Fig. 5:**
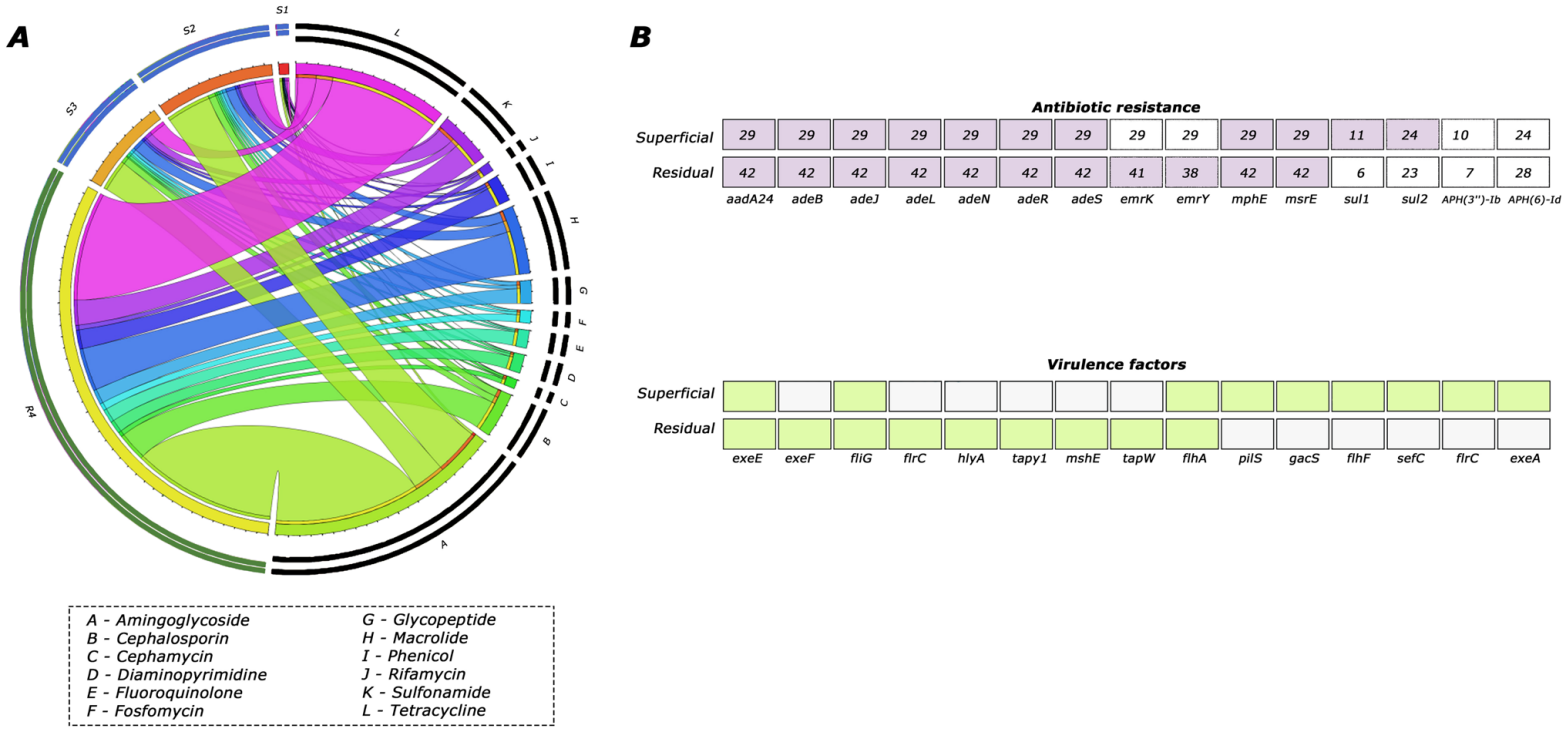
Molecular markers detected from Metagenome-assembled genomes. **A** Relationship between the frequency of genes coding for the most common drug-associated molecular markers at each sampling point (S1–S3 surface water samples, R4 = wastewater samples). **B** Molecular markers associated with resistance (upper panel); molecular markers associated with virulence factors (lower panel)

Molecular markers associated with antibiotic resistance were screened by comparison with the CARD database. Our research revealed the ten most frequently detected molecular markers related to drugs in wastewater and surface water samples. In the wastewater samples, indicated by dark green in the outer ring (R4), we found that the most common antibiotic resistance molecular markers, shown by their wide bands in the circus (Fig. 4A), were associated with tetracyclines, aminoglycosides, and macrolides. Conversely, in the surface water samples (S1–S3), marked in light blue on the outer ring, aminoglycosides and tetracycline consistently dominated. Additionally, sulfonamides were frequently observed at points S1 and S3. Reflecting the trends in wastewater, macrolides were most prominent at point R4, following the other two drugs (Fig. 5A).

Moreover, we selected the top 15 molecular markers frequently detected in both wastewater and surface water samples. In the upper panel marked in pink in Fig. 5B, the markers associated with antibiotic resistance can be observed. Each marker's frequency is indicated, with a maximum frequency of 29 for surface waters and 42 for wastewater. This emphasizes the prevalence of markers *ermK* and *ermY* in surface waters compared to sewage. In contrast, sul1 and sul2 were more common in wastewater than in surface waters. Regarding the molecular markers associated with virulence factors, located in the lower panel marked in green, we found that markers *hlyA*, *mshE*, and *flrC* were more abundant in wastewater than in surface waters. Conversely, *gacS* and *sefC* were predominant in surface waters but absent in sewage.

While moving from the outermost to the innermost part of MAGs, the antibiotic resistance markers in red that align with CARD can be noticed. The reference genomes for each species can be found in purple, and in gray, the assembled genome from the study, along with its respective contigs, can be seen. Our study successfully identified various microbial species. For instance, we detected *Acinetobacter johnsonii* in six different genome bins. When we examined its genome, we found 939 contigs and three molecular markers in CARD. Similarly, we identified *Megamonas funiformis* in two genome bins, revealing a genome consisting of 505 contigs and one molecular marker in CARD.

Additionally, the genome of *Phocaeicola vulgatus*, identified in three bins, comprised 689 contigs and four molecular markers in CARD. Our study also revealed the presence of other species, including *Aliarcobacter cryaerophilus_A*, *Sphaerotilus montanus, Prevotella copri, Aeromonas media, Aeromonas rivipollensis, Bacteroides uniformis*, and *Phascolarctobacterium_A_succinatutens*. Furthermore, through analyses using high-quality WGS data, we assigned the following species: *Kaistella chaponensis, Trichococcus flocculiformis, Zoogloea ramigera, Alistipes putredinis, Lactococcus A raffinolactis, and Faecalibacterium prausnitzii.*

Also, we identified antimicrobial resistance markers. *Acinetobacter johnsonii,* for instance, exhibited 11 molecular markers associated with the beta-lactam resistance pathway, six genes linked to the Vancomycin resistance pathway, and five genes involved in the Cationic antimicrobial peptide (CAMP) pathway. Similarly, in *Megamonas funiformis*, we identified five genes within the beta-Lactam pathway, five in the Vancomycin pathway, and 5 in the CAMP pathway.

### Molecular Markers of Interest in Health Associated with MAGs

**Fig. 6:**
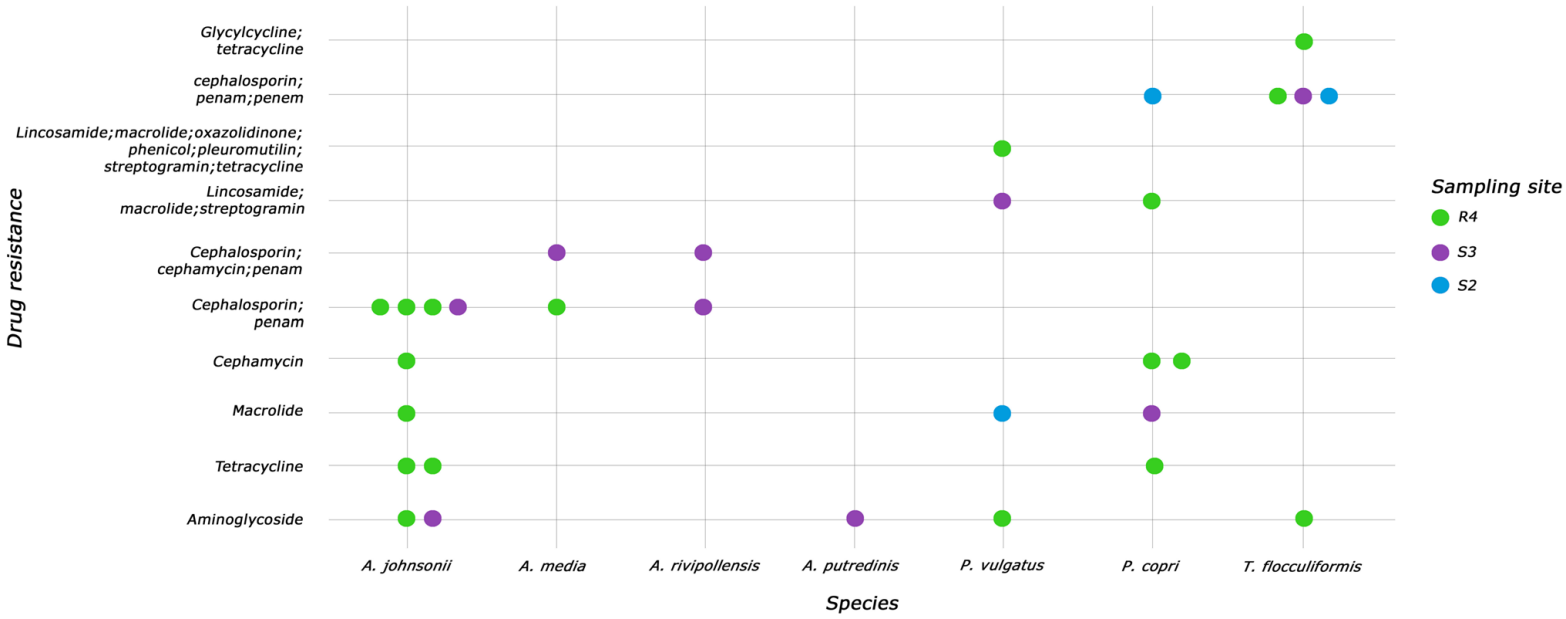
Molecular health-related markers associated with the MAGs for each species. Each point represents an assembled genome of a specific species

Furthermore, using the previously obtained assemblages, we identified molecular markers related to health, specifically those associated with antibiotic resistance and virulence factors in the MAG species. We categorized resistance markers by species and their respective sampling points. This approach helped us identify the molecular markers most abundant within each species. In Fig. 6, genome assemblies for each species can be observed, depicted as circles. The color reflects the source of each assembly, indicating whether the sample originated from wastewater or surface water and, in the latter scenario, the specific sampling point. The frequently detected antimicrobial resistance markers in wastewater (R4) included aminoglycoside, tetracycline, cephamycin, and cephalosporin, each found in three species.

In contrast, in surface waters (S3, S2), resistance markers for cephalosporins were identified in five species, while that for macrolide appeared in two. Furthermore, we identified virulence factors exclusively found in the species *Zoogloea ramigera*. In this case, we detected the genes *hsiC1/vipB*, *hsiC1/vipA*, and *pilT*.

## Discussion

Studying the microbiome in aquatic environments is essential to understanding the microorganisms and their interactions, especially those that could impact public health [50]. From the findings of the physicochemical analyses, we can draw several conclusions. First, the elevated nitrite values in surface waters indicate contamination related to agricultural activities [51]. In contrast, the high values of temperature, suspended solids, and BOD in wastewater imply greater contamination, primarily due to domestic, hospital, and stormwater contaminants [52–54]. The identification of parasites like *Giardia* spp. and *Cryptosporidium* spp. aligns with findings from other studies. These parasites have been detected in various regions, including Spain in wastewater [55], Poland in surface waters [56], and within the Nariño (Colombia), as reported by Sánchez [61]. This underscores the concern associated with reusing surface water, as water serves to transmit these parasites [57]. These findings emphasize the potential public health risks and draw attention to the possible impact of the Juan XXIII wastewater collector, which discharges untreated water into surface waters.

Our study aimed to identify the most abundant families at each collection point to understand how they change over time. When examining the families present at the S3 surface water point (Figs. 1, S2), we noticed the widespread distribution of the Bacteroidaceae family in the environment. This is attributed to their large genomes, which enable them to adapt to diverse conditions [58]. Nevertheless, it is worth noting that some members of this family can act as opportunistic pathogens, potentially causing human infections. This suggests that there was likely a higher load of contaminants linked to mammalian excretions in September, as some of the bacterial species identified from these families thrive in the gastrointestinal system of particular mammal [59].

Similarly, we observed significant changes in the Rhodocyclaceae family. Members of this family have been identified in aquatic environments, as demonstrated by Chernitsyna et al. They also found that these family members play a role in bioremediation, particularly in denitrification processes. This aligns with our research, as we detected elevated levels of nitrites in surface waters, suggesting the possible involvement of these species in denitrification processes [60]. We also observed a similar pattern in the Pseudomonadaceae family. While some species are commonly found in the environment, it is essential to highlight the presence of pathogenic species that could pose public health concerns. The findings in surface waters provide valuable insights into identifying bacteria present in the environment, particularly emphasizing bacterial families that include members with pathogenic potential. This underscores the importance of monitoring this environment to comprehensively understand the associated risks when in contact with this valuable resource.

Regarding the families identified at wastewater sampling point R4 (Figs. 1, S3), we observed significant variations in the abundance of all families across different months. The Burkholderiaceae family exhibited higher abundance in September, suggesting increased contamination from hospital, domestic, and industrial wastewater sources [61]. We also noticed fluctuations in the Rhodocyclaceae family, which has been previously reported in wastewater treatment systems by Wang and colleagues. They identified some members of this family as participating in denitrification processes [62]. Another noteworthy finding was the higher abundance of the Campylobacteraceae family in February. This finding is significant as certain family members are associated with diarrheal diseases, indicating the presence of relevant pathogens [63].

Furthermore, identifying families such as Comamonadaceae and Moraxellaceae highlights the importance of monitoring the microbiome. Some members of these families have been reported as opportunistic pathogens. However, it is essential to note that they also include species with potential applications in biotechnology due to their advantages [64]. The Enterobacteriaceae family comprises members commonly found in the intestines of animals and humans. Fluctuations in the abundance of this family across different months may be linked to variations in the volume of wastewater discharges from homes and hospitals [65]. Finally, the Shewanellaceae family predominantly appeared exclusively at point R4. Considering the characteristics of this family, its presence at the wastewater point is expected, as some of its members are typically found in aquatic environments and play a fundamental role in the decomposition of organic matter [66]. This information emphasizes the diverse array of wastewater bacteria, including pathogenic and beneficial organisms. Furthermore, it emphasizes the concern that discharging wastewater into the Pasto River might be causing a detrimental impact on the environment.

Additionally, it is crucial to highlight species of particular interest, particularly those that may represent a risk to public health due to their pathogenic (Fig. 2A). Our research identified *Bacteroides cellulosilyticus*, widely distributed in the human intestine [67], suggesting fecal contamination in the water. Furthermore, *Raoultella ornithinolytica*, known to be found in bodies of water and even in drinking water systems, as demonstrated by Zou et al. [68], raises concerns as this species has been associated with hospital-acquired infections and in immunosuppressed individuals [68]. Equally important to note is *Pseudomonas aeruginosa*, a species that is not only found in a wide range of environments, including hospitals and sewage systems but also represents a significant public health concern as a nosocomial pathogen and a multi-resistant to antibiotics [69]. The most abundant species identified was *Aeromonas media*, a bacterium capable of causing various diseases in both humans and animals, and it has also been reported to exhibit resistance to specific antibiotics [70]. Lastly, we identified the *Burkholderia cepacia* complex, a group of bacteria widely distributed and highly adaptable, with a tendency for rapid genetic and phenotypic mutations. Despite their environmental origin, these species are of concern as opportunistic pathogens [71]. The identification of these species provides us with valuable tools for regulating the use of water resources and estimating potential risks associated with their presence. It also emphasizes the critical importance of continuously monitoring this resource, as this surveillance is essential for ensuring the control and prevention of public health emergencies. Water contaminated with specific microorganisms can serve as a means of propagating these pathogens, potentially leading to severe illnesses.

Additionally, we would like to highlight species with notable beneficial potential (Fig. 2B), such as *Thauera sp. MZ1T* is distinguished by its unique ability to produce a specific exopolysaccharide, consequently improving the viscosity of its environment. This distinctive feature significantly improves the efficiency of disinfection processes in industrial wastewater treatment plants [72]. In contrast, we have also identified *Alicycliphilus denitrificans,* capable of biodegrading a wide range of organic contaminants, suggesting its potential to remediate xenobiotic substances, particularly in contaminated water bodies [73]. Furthermore, among the most abundant species, we have identified *Polaromonas naphthalenivorans,* a bacteria reported in various water bodies that can decompose naphthalene, a common component in fuels, pesticides, and other substances [74, 75]. Our findings go beyond simply identifying species with promising attributes for applications in biotechnology, including bioremediation and the decomposition of harmful compounds. They also shed light on species with potential biotechnology applications and provide valuable information on contaminants that may be present in both wastewater and surface water. This underlines the critical importance of recognizing and characterizing microbial species with substantial potential in these environments.

Additionally, we identified archaea in surface waters, including *Methanobrevibacter smithii*, which is prevalent in the human intestine [76], and *Methanococcus maripaludis*, a marine methanogenic organism [77]. In wastewater, we found *Methanosarcina barkeri*, which has been widely studied as a model of methanogenesis [78]. Regarding viruses, we identified the *Aeromonas virus phiO18P viruses,* known for their ability to transmit resistance and virulence [79], and the *Acanthamoeba polyphaga mimivirus*, responsible for infecting amoebas [80]. Our research highlights a diverse spectrum of microorganisms in wastewater and surface water, some associated with human and animal fecal matter, indicating contamination. We recommend continuing such analyses and improving public databases to collect more complete information on all microorganisms, including parasites and fungi. It would also be highly beneficial to increase the sequencing capacity by including more gigabytes (Gb) of data. This would provide a deeper understanding of the eukaryotic communities, as most of the data is currently allocated to bacterial communities due to their significant diversity.

The generation of metagenome-assembled genomes—MAGs, played a crucial role in identifying previously unknown microorganisms. In environmental samples, MAGs provide essential information about the environment, specific pressures, and the potential to understand non-cultivable or challenging-to-isolate species [89]. This study identified a diverse range of bacterial species, including some originating from the intestine, gastrointestinal system, and feces. These species include *Phocaeicola vulgatus* [90], *Megamonas funiformis* [91], *Prevotella copri* [92], *Bacteroides uniformis* [90], *Phascolarctobacterium_A_succinatutens* [93], *Alistipes putredinis* [94], *Faecalibacterium prausnitzii* [95] y *Aliarcobacter cryaerophilus_A* [96]. Additionally, species commonly found in water bodies, biofilms, and related environments were detected, such as *Acinetobacter johnsonii* [97], *Sphaerotilus montanus* [98], *Aromonas media* [99], *Aeromonas rivipollensis* [100], *Kaistella chaponensis* [101], *Trichococcus flocculiformis* [102] y *Zoogloea ramigera* [103]. Finally, *Lactococcus A_raffinolactis* is distributed in fermented foods [104]. These findings illustrate the diverse range of genomes within the assembled bacterial species and their respective origins. They contribute to a global understanding that the dominant species originate from the digestive system and those commonly found in the environment.

A key focus of our research centers on the genome of *A. johnsonii*, a microorganism widely distributed in aquatic environments yet also reported as an opportunistic pathogen. Additionally, we note that the category "Information storage and processing" predominates when examining its functional characteristics. This indicates that this species primarily allocates its coding potential to J (translation, ribosomal structure, and biogenesis) and L (replication, recombination, and repair) [47]. Furthermore, assembling the genomes of *Megamonas funiformis* and *Phocaeicola vulgatus* provides a valuable tool to enhance our comprehension of their functions in the human intestine [90, 91]. Regarding functional characteristics (Fig. 3), *P. vulgatus* displays a higher frequency of COGs related to "cell processing and signaling," particularly in M (cell wall/membrane/envelope biogenesis). This underscores the crucial role of this process in maintaining and protecting these species within their environment. *M. funiformis* directs its coding potential toward “metabolism.” All of this contributes to a better understanding of the sets of genes that could potentially impact the adaptation and survival of these species in aquatic environments [105, 106]. At a broader level, the most frequent categories were "Unknown function" and "Unclassified," underscoring the importance of exploring and contributing information to public databases to obtain more profound knowledge about these functional attributes. Finally, it is essential to note that genes associated with antibiotic resistance pathways have been identified in all three species, including beta-lactam resistance, vancomycin resistance, and cationic antimicrobial peptide (CAMP) resistance [47]. Therefore, we encourage further comprehensive studies on these functional characteristics, with a specific focus on identifying the precise functions of each gene within these pathways. This effort will allow us to provide comprehensive information on potential risks and shed light on the acquisition of resistance by certain pathogenic species that currently lack detailed characterization (Figs. S4, 3).

Comparative genomics analysis provides valuable insights into the relationships among the samples (Fig. 4). When we conducted the pangenome analysis for the species *A. johnsonii*, we observed that reference genome 6 is not clustered with the other reference genomes and the assembled genomes from the study. This could be attributed to the percentage of the core genome identified in this study, which amounts to 218 genes, while the accessory genome accounts for 12.958 genes out of a total of 13.176 genes. This hypothesis is proposed because the reference genome was isolated from water in 2019 [110], and its extensive array of accessory genes might have led to its distinct separation from the others. Furthermore, this species is characterized by having high plasticity in the genome [97].

On the contrary, *M. funiformis* displayed a cluster of diverse samples with no significant grouping differences. However, it is worth noting that this species has a limited number of reference genomes. Lastly, in the case of *P. vulgatus*, sample DP46 from the study appeared distinctly separated from the other samples. This sample was collected from point R4 in wastewater. Nevertheless, it is essential to acknowledge that this genome might be highly fragmented, and its assignment may only partially be accurate. Besides, it may also be influenced by selection pressures such as antibiotic resistance. Likewise, it is essential to highlight the importance of understanding this species since, although it is part of the human intestine, it has stood out for being an opportunistic pathogen [111].

Rivers have encountered significant challenges due to human activities [82, 83], and one of these challenges is pollution caused by the improper disposal of antibiotics. Antibiotics are considered emerging contaminants because they cannot be broken down, leading to their accumulation in the aquatic environment and the development of selection pressure [84, 85]. We examined molecular markers associated with antibiotic resistance (Fig. 5), identifying a high relative abundance related to tetracyclines and aminoglycosides in wastewater. This pattern is consistent with the global context, as both types of drugs are widely used in human, animal, and agricultural healthcare [84]. Furthermore, we noticed a substantial frequency of genes conferring resistance to macrolides, likely linked to their increasing use in livestock and agriculture. As a result, the aquatic environment has become a conduit for the spread of antibiotic-resistant genes [86]. Conversely, in the surface waters of the Pasto River, we identified a high prevalence of genes associated with aminoglycoside and tetracycline resistance, which underscores the potential impact of wastewater discharge directly into surface waters. At point S1, we frequently detected sulfonamide resistance genes, suggesting a notable contribution to the aquatic environment from contaminants such as urine and feces from humans and animals due to their use in medical treatment [87].

In the other analysis, we identified specific molecular markers in wastewater and surface water (Fig. 4). One of these markers is *EmrK*, responsible for conferring resistance to tetracyclines [81], and *EmrY*, which confers resistance to macrolides, streptogramins, and lincosamides. *Emr* markers are typically found on plasmids, facilitating horizontal gene transfer and enhancing the transmission of these genes to other pathogens [35, 82]. This finding aligns with our previous results, where we observed a high prevalence of genes that confer resistance to tetracyclines. In the case of surface waters, we observed a higher frequency of the *sul1* and *sul2* markers, which are responsible for conferring resistance to sulfonamides. This corresponds with our previous findings, where we identified the gene associated with sulfonamide resistance as commonly present in surface waters [35]. Notably, both markers have been reported in bacterial genera, such as *Acinetobacter*, *Aeromonas*, and *Pseudomonas*, which were also abundant in our study.

Furthermore, identifying molecular markers associated with virulence factors allows us to understand the enzymes, toxins, and other elements that enable pathogenic microorganisms to cause more harm [83]. For instance, we detected markers like *hlyA* [84] in wastewater, which can damage the cell membrane through exotoxin. We also found markers like *mshE* [85], which modify the pilus to enhance adhesion for colonization in aquatic environments and biofilm formation. Additionally, we identified the flagellar transcriptional activator gene *flrc* [86]. In surface waters, we found markers that regulate virulence factors related to stress tolerance, pathogen motility, and more, such as *gacS* [87]. We also identified markers like *sefC* [88], which facilitate the adhesion and colonization of various substrates and cells. While identifying microorganisms in water bodies is crucial, detecting the molecular markers that enhance their virulence, and potential harm is equally essential.

Identifying antibiotic resistance molecular markers from predominant species (Fig. 6), particularly those conferring resistance to aminoglycosides and tetracyclines, raises concerns about the potential public health risks associated with some species, mainly because they are classified as opportunistic pathogens. Moreover, the detection of genes related to resistance to cephamycins, a drug used in the treatment of infections [107], in addition to cephalosporins (which constitute approximately 50% of the antibiotics used in human health) in wastewater, is a cause for concern. This implies the creation of selective pressure in this environment and suggests fecal contamination in surface waters [108]. These findings highlight the inadequate elimination and accumulation of these drugs in water [109]. Regarding markers associated with virulence factors, they were only identified in the genome of *Zoogloea ramigera*. Within this genome, we identified genes such as *hsiC1/vipB* and *hsiC1/vipA*, responsible for tubule-forming proteins of the type VI secretion system *VipB* and type VI *VipA*. *PilT* is associated with the sporadic motility protein PilT [36]. In conclusion, while these analyses provide an initial perspective on markers in the aquatic environment, conducting more comprehensive studies in future research is advisable to obtain robust information for resource monitoring and surveillance.

## Conclusion

This study provides vital information about the current state of the Pasto River. We comprehensively analyze the river and its wastewater, explicitly focusing on evaluating physicochemical and microbiological parameters. In essence, this pioneering study is of great importance to the region as it emphasizes the importance of continuous monitoring and surveillance of this critical resource, especially in the context of possible water reuse. Furthermore, this study represents the first microbiological characterization at a regional level, covering both taxonomic and functional aspects, made possible thanks to next-generation sequencing techniques, which provide valuable information on its relevance to public health. This contributes to informed decision-making by relevant authorities responsible for managing these water resources. However, we recommend that future studies incorporate complementary techniques to evaluate the viability of these microorganisms circulating in these bodies of water using next-generation sequencing techniques. Furthermore, paying attention to traditional physicochemical and microbiological analyses is essential to correlate these results with sequencing data. Finally, we encourage the scientific community to continue with the characterization studies of water bodies, and thus nourish public databases to identify and characterize even more microorganisms in future studies.

## Supplementary Information

Below is the link to the electronic supplementary material.

Supplementary file1 (PDF 433 KB)

Supplementary file2 (DOCX 712 KB)

Supplementary file3 (PDF 5513 KB)

Supplementary file4 (PDF 2838 KB)

Supplementary file5 (PDF 8191 KB)

Supplementary file6 (EXCEL 16 KB)

Supplementary file7 (EXCEL 162 KB)

Supplementary file8 (EXCEL 2354 KB)

Supplementary file9 (EXCEL 3256 KB)

Supplementary file10 (DOCX 20 KB)

## Data Availability

The data obtained and analyzed in this study are available in the European Nucleotide Archive (ENA) repository under project number PRJEB64340.
