# Supplementary material for: Metagenomic Analysis of Surface Waters and Wastewater in the Colombian Andean Highlands: Implications for Health and Disease
Source: Curr Microbiol. 2025 Feb 28;82(4):162. doi: 10.1007/s00284-024-04019-7 (PMC11870934; doi:10.1007/s00284-024-04019-7)
Supplement: Supplementary file 1 — Supplementary file1 (PDF 433 KB) [file 284_2024_4019_MOESM1_ESM.pdf]

Current Microbiology

AUTHOR'S CHECKLIST FOR MANUSCRIPT SUBMISSION

A copy of this checklist should accompany each submitted paper.

Your manuscript must be prepared according to the Instructions for Authors which are available online and accessible here.

General

☒ **At least the corresponding author has read carefully the Instruction for Authors guide.**

☒All authors have read the submitted version of the paper AND have approved its Submission.

☒ All authors' names have been provided as 'full first name, (middle initial), full last name'

☒ All authors have double checked their affiliation (with precise location).

☒The contact information for the corresponding author(s) is as follows:

- o Provided an Institutional email/ORCID number whenever possible
- o Institutional phone number

☒ All text, including abstract, tables, references, footnotes and figure legends, is double-spaced printed. All lines and pages are numbered.

Compliance with ethical standards

☒ Authors' contribution statement has been included.

☒ A disclosure of potential conflict of interest has been included.

☐ Ethical approval for research involving human participants and/or animals: if applicable

☐ Informed consent has been provided: if applicable (human participants)

Abstract

☒ The abstract is clear and concise and summarizes the content of the paper without presenting extensive experimental details.

- Length (250 words max)
- Structure: one paragraph without subheadings
- Keywords (5-7)

Manuscript

☒ The English language usage has been checked by a fluent English speaker and/or a professional language editing service.

☐ The length of the manuscript is according to Instruction for authors taking into account the article type.

☒ Abbreviations are defined at first mentioning.

☐ Figures have been submitted electronically separately from the main manuscript file, at high resolution and preferentially in TIFF or EPS.

☒ Figure captions summarize the result or major of the data you are presenting in the figure with a brief description of the methods necessary to understand the figure without having to refer to the main text.

☒ The submission has no more than five main figures with up to four panels each (Original paper) or up to two figures with up to four panels each (Short Communications).
☒ Reference formatting is according to the formatting outlined in the instructions for authors.
☐ The number of references is according to the article type.

Data
☐ Raw data/original images of uncropped and non-adjusted gels/blots should be available to be provided as supporting material upon request.
☒ The sequence accession number(s) have been provided at the end of Material & Methods section.

New taxa
☐ The type strains for new taxa have been deposited in at least two different culture collections
☐Full (required for prokaryotes) or draft genome sequencing data is provided.

for fungi see: https://doi.org/10.1186/s43008-021-00063-1
for prokaryotes see:https://doi.org/10.1099/ijsem.0.000778

Microbial isolates
☐Microbial isolates should be correctly identified and characterized using molecular methods.
☐In case of 16S rRNA gene sequence comparison (more than 1000 nt) is mandatory between prokaryotic isolates and type strains of validly named species.
☐ The isolate identification was performed following the Submission guidelines (Microorganism Identification section) and recommendations published in this Editorial.

Statistics
☐ A clear experimental design with the treatments and replicates clearly described has been included.
☐ The F values, the degrees of freedom and the precise p values obtained have been calculated.
☐ The assumptions of the statistical test have been tested.
☒ Statistical information (number of replicates, asterisks denoting P-values, statistical tests) has been provided.

Suggested Reviewers
☐ Suggested reviewers are from different countries and different institutions than the submitting authors.
☐ The reviewer candidates have a verifiable email address and affiliation.

Springer Nature Editorial Policies
☐ The manuscript presented adheres to the Editorial Policies of Springer Nature.
All policies, also including our preprint policy are accessible here: https://www.springernature.com/gp/policies/editorial-policies
