# Supplementary material for: Metagenomic Analysis of Surface Waters and Wastewater in the Colombian Andean Highlands: Implications for Health and Disease
Source: Curr Microbiol. 2025 Feb 28;82(4):162. doi: 10.1007/s00284-024-04019-7 (PMC11870934; doi:10.1007/s00284-024-04019-7)
Supplement: Supplementary file 2 — Supplementary file2 (DOCX 712 KB) [file 284_2024_4019_MOESM2_ESM.docx]

**Supplementary material**

**Fig. S1.** Schematic representation of A) Model implemented for collecting wastewater and surface water samples from pedestrian and vehicular bridges B) System used for a sample collection from a simple collection and composite collection for wastewater and surface water.

**Fig. S2.** Boxplot diagram illustrating families at point S3 with statistically significant differences (P < 0.05)*.

**Fig. S3.** Boxplot diagram illustrating families at point R4 with statistically significant differences (P ≤ 0.001)***.
