# Supplementary figures and images for: Metagenomic Analysis of Surface Waters and Wastewater in the Colombian Andean Highlands: Implications for Health and Disease
Source: Curr Microbiol. 2025 Feb 28;82(4):162. doi: 10.1007/s00284-024-04019-7 (PMC11870934; doi:10.1007/s00284-024-04019-7)

**A**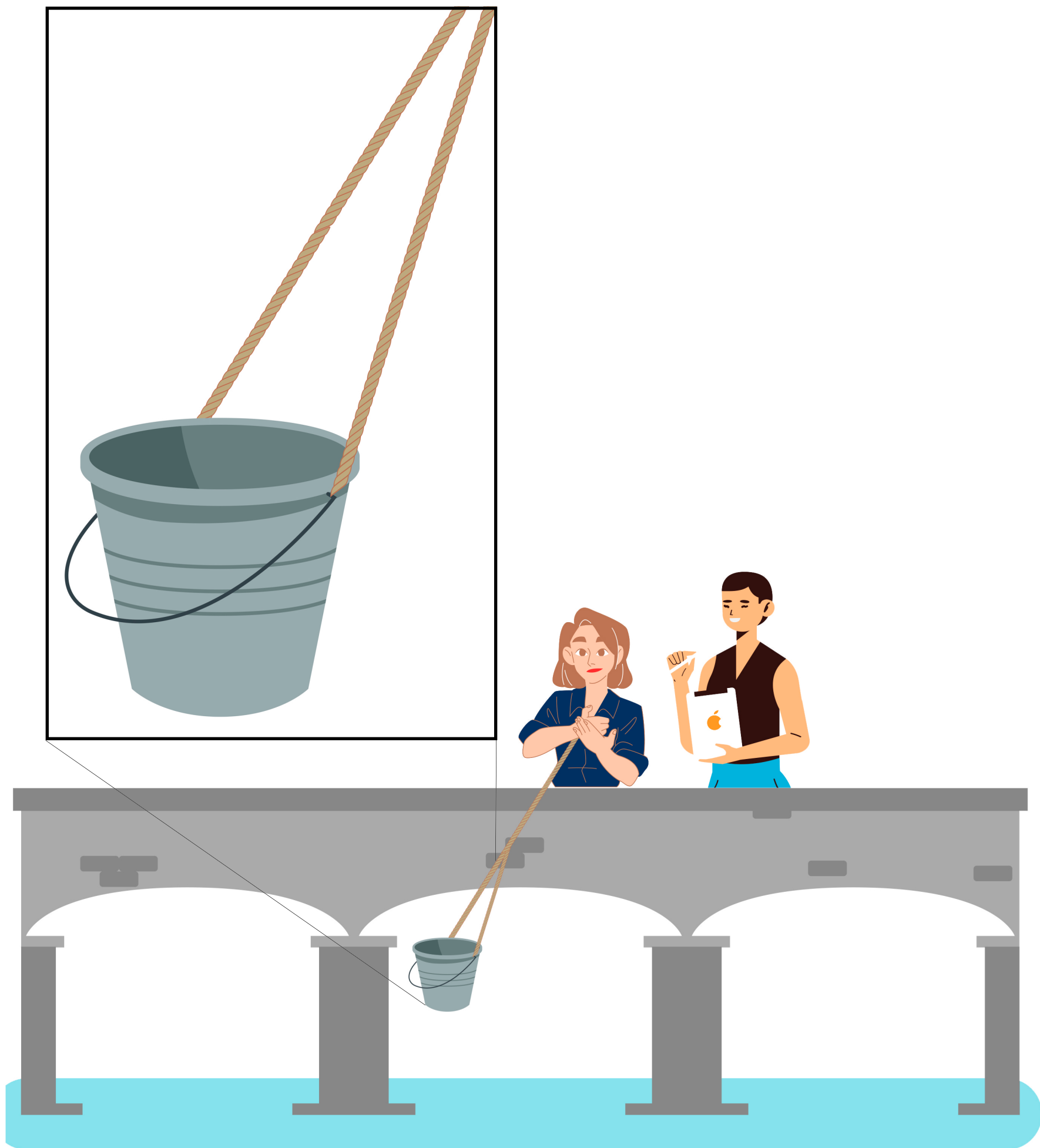**B**

Surface water

Single Sample

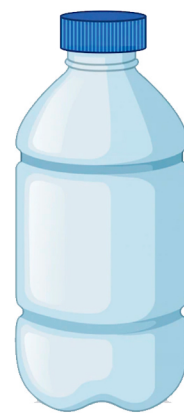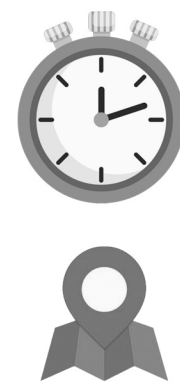

Composite sample

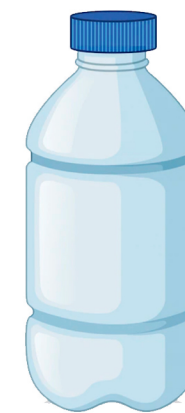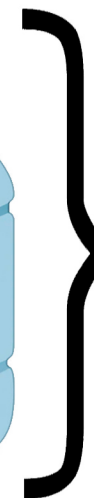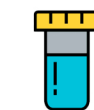

9:00

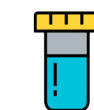

9:15

15 min

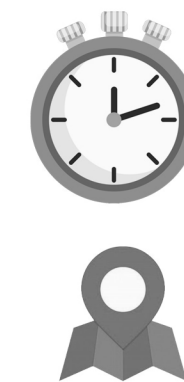

Wastewater

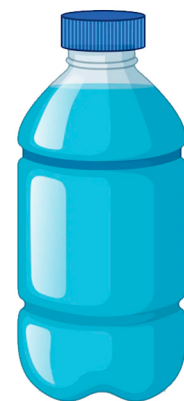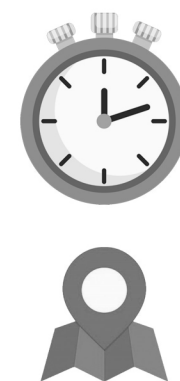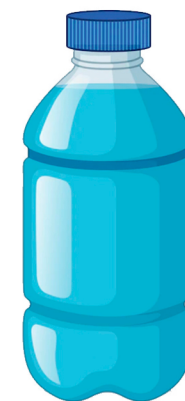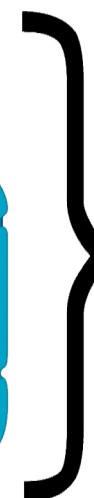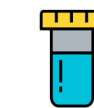

7:00

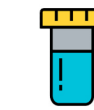

8:00

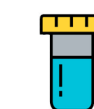

9:00

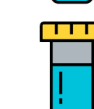

10:00

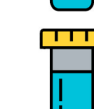

11:00

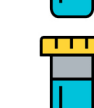

12:00

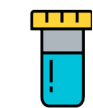

13:00

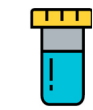

14:00

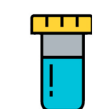

15:00

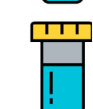

16:00

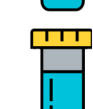

17:00

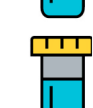

18:00

60 min

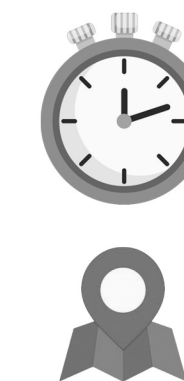

Supplement: Supplementary file 3 — Supplementary file3 (PDF 5513 KB) [file 284_2024_4019_MOESM3_ESM.pdf]

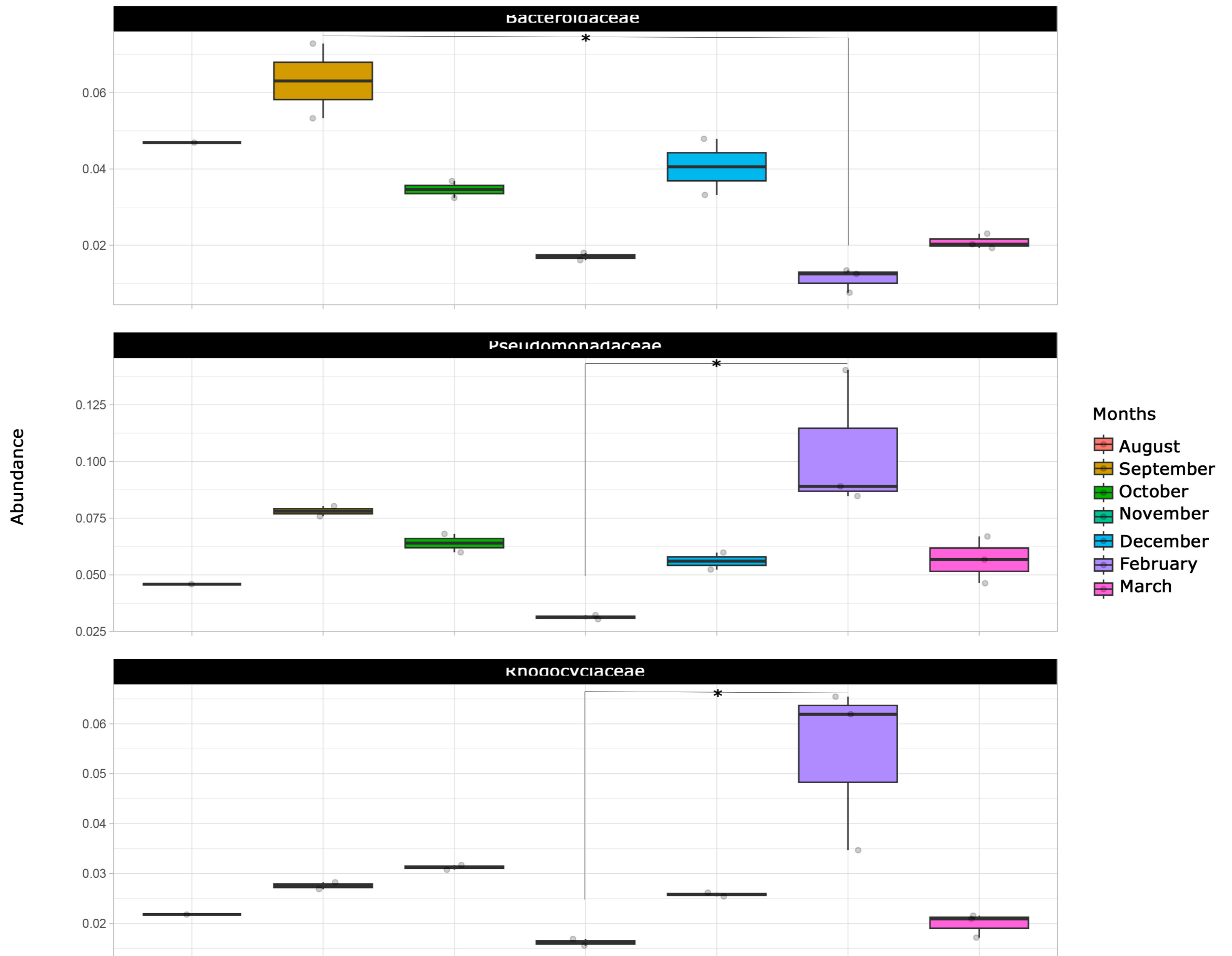

Supplement: Supplementary file 4 — Supplementary file4 (PDF 2838 KB) [file 284_2024_4019_MOESM4_ESM.pdf]

Abundance

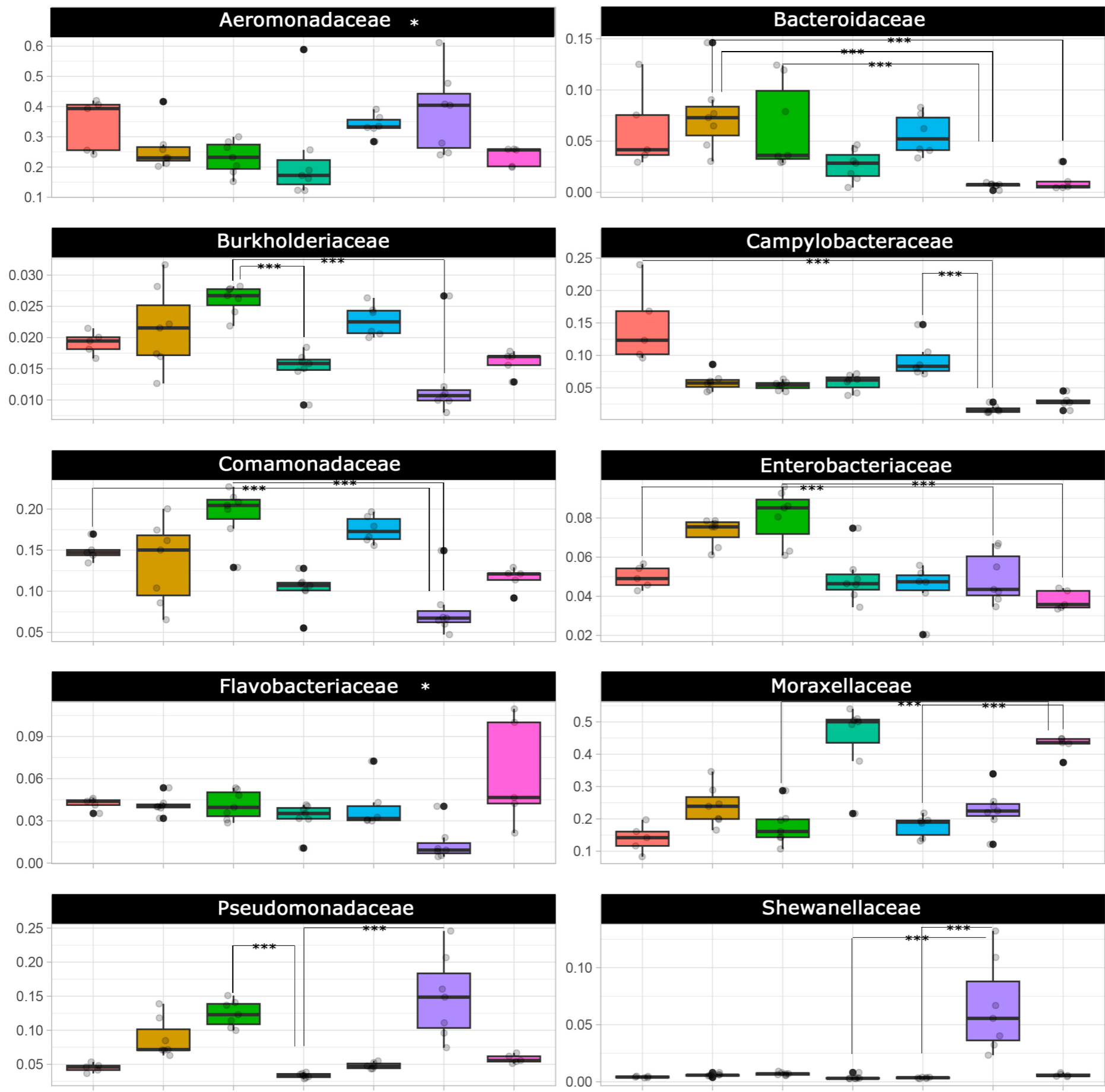

Supplement: Supplementary file 5 — Supplementary file5 (PDF 8191 KB) [file 284_2024_4019_MOESM5_ESM.pdf]
