# Supplementary material for: Metagenomic Analysis of Surface Waters and Wastewater in the Colombian Andean Highlands: Implications for Health and Disease
Source: Curr Microbiol. 2025 Feb 28;82(4):162. doi: 10.1007/s00284-024-04019-7 (PMC11870934; doi:10.1007/s00284-024-04019-7)
Supplement: Supplementary file 10 — Supplementary file10 (DOCX 20 KB) [file 284_2024_4019_MOESM10_ESM.docx]

| **Specie** | **Genbank Accessions** | |
| --- | --- | --- |
| *Acinetobacter johnsonii* | 1 | CP119256, CP119255, CP119257 |
|  | 2 | CP119200, CP119201 |
|  | 3 | CP090416, CP090417 |
|  | 4 | CP090180, CP090181 |
|  | 5 | CP083947, CP083948, CP083949 |
|  | 6 | CP079748, CP079749, CP079750 |
|  | 7 | CP070866, CP070867, CP070868, CP070869 |
|  | 8 | CP068206, CP068202, CP068203, CP068204, CP068205, CP068207, CP068208, CP068209, CP068210 |
|  | 9 | CP068195, CP068196, CP068197, CP068198, CP068199, CP068200, CP068201 |
|  | 10 | CP068187, CP068185, CP068186, CP068188, CP068189, CP068190, CP068191, CP068192, CP068193, CP068194 |
|  | 11 | CP065666, CP065663, CP065664, CP065665 |
|  | 12 | CP059080 |
|  | 13 | CP045103, CP045104, CP045105, CP045106, CP045107 |
|  | 14 | CP045101, CP045102 |
|  | 15 | CP045099, CP045100 |
|  | 16 | CP045051 |
|  | 17 | CP043307, CP043308, CP043309, CP043310 |
|  | 18 | CP037424, CP037425 |
|  | 19 | CP031011 |
|  | 20 | CP022298, CP022299, CP022300, CP022301, CP022302 |
|  | 21 | CP010350, CP010352, CP010353, CP010354, CP010355, CP010356, CP010357, CP010358, CP010351 |
| *Aeromonas media* | 1 | CP118993, CP118994, CP118995, CP118996 |
|  | 2 | CP075564, CP075565 |
|  | 3 | CP070623 |
|  | 4 | CP067417, CP067418, CP067419 |
|  | 5 | CP043579 |
|  | 6 | CP038441, CP038442 |
|  | 7 | CP038443 |
|  | 8 | CP038444 |
|  | 9 | AP022188 |
| *Aeromonas rivipollensis* | 1 | CP027856 |
| *Aliarcobacter cryaerophilus_A* | 1 | CP026655 |
|  | 2 | CP026656 |
|  | 3 | CP060264 |
|  | 4 | CP060692 |
|  | 5 | CP060693 |
|  | 6 | CP060694 |
| *Alistipes putredinis* | 1 | CP091730 |
| *Bacteroides uniformis* | 1 | CP054204, CP054199, CP054200, CP054203, CP054202, CP054201 |
|  | 2 | CP102263 |
|  | 3 | CP083677 |
|  | 4 | CP103250, CP103251, CP103252 |
|  | 5 | CP072255, CP072256 |
|  | 6 | CP102263 |
|  | 7 | CP072220, CP072221, CP072222, CP072223 |
|  | 8 | CP072236, CP072237, CP072238 |
|  | 9 | CP072239, CP072240, CP072241 |
|  | 10 | CP065889 |
| *Megamonas funiformis* | 1 | AP024966 |
|  | 2 | CP048627, CP048628 |
|  | 3 | CP084018 |
| *Phascolarctobacterium_A succinatutens* | 1 | CP097284 |
| *Phocaeicola vulgatus* | 1 | CP000139 |
|  | 2 | CP103067 |
|  | 3 | CP103197, CP103198 |
|  | 4 | CP096965, CP096968, CP096966, CP096967 |
|  | 5 | CP081912, CP081909, CP081910, CP081911 |
|  | 6 | CP043529 |
|  | 7 | CP013020 |
|  | 8 | CP072234, CP072235 |
|  | 9 | CP068488 |
|  | 10 | CP068489, CP068490 |
| *Prevotella copri* | 1 | CP102288 |
|  | 2 | CP042464, CP042465 |
|  | 3 | CP102288 |
|  | 4 | CP085932, CP085933, CP085934 |
| *Kaistella chaponensis* | 1 | FTOI00000000 |
| *Lactococcus_A raffinolactis* | 1 | CALL00000000 |
|  | 2 | VBTC00000000 |
| *Sphaerotilus montanus* | 1 | JACCFH000000000 |
|  | 2 | JACCPZ000000000 |
| *Zoogloea ramigera* | 1 | VMEK00000000 |
|  | 2 | BJNV00000000 |
| *Faecalibacterium prausnitzii* | 1 | OX636702 |
|  | 2 | CP117963 |
|  | 3 | CP107213 |
|  | 4 | CP065381 |
|  | 5 | CP065377 |
|  | 6 | CP065376 |
|  | 7 | CP065382 |
|  | 8 | CP065380 |
|  | 9 | CP065378 |
|  | 10 | CP065379 |
|  | 11 | [CP048437](http://www.ncbi.nlm.nih.gov/nuccore/CP048437) |
|  | 12 | LR699017 |
|  | 13 | CP030777 |
|  | 14 | CP030777 |
|  | 15 | CP022479 |
|  | 16 | CP023819 |
|  | 17 | FP929046 |
|  | 18 | FP929045 |
| Trichococcus flocculiformis | 1 | JAAZCD000000000 |
|  | 2 | FJMZ00000000 |
|  | 3 | FQVR00000000 |
|  | 4 | FOQC00000000 |
